# Identification of host gene-microbiome associations in colorectal cancer patients using mendelian randomization

**DOI:** 10.1186/s12967-023-04335-9

**Published:** 2023-08-10

**Authors:** Yaoxian Xiang, Chan Zhang, Jing Wang, Yurong Cheng, Li Wang, Yingying Tong, Dong Yan

**Affiliations:** Department of Oncology, Beijing Luhe Hospital Affiliated to Capital Medical University, Beijing, 101149 China

**Keywords:** Mendelian randomization (MR), Gut microbiota, Gene, Colorectal cancer (CRC), Causal relationship

## Abstract

**Background:**

There are many studies indicating that alterations in the abundance of certain gut microbiota are associated with colorectal cancer (CRC). However, a causal relationship has not been identified due to confounding factors such as lifestyle, environmental, and possible reverse causal associations between the two. Furthermore, certain host gene mutations can also contribute to the development of CRC. However, the association between genes and gut microbes in patients with CRC has not been extensively studied.

**Methods:**

We conducted a two-sample Mendelian randomization (MR) study to reveal the causal relationship between gut microbiota and CRC. We obtained SNPs associated with gut microbiome abundance as instrumental variables (IVs) from a large-scale, multi-ethnic GWAS study, and extracted CRC-related datasets from an East Asian Population genetic consortia GWAS (AGWAS) study and FinnGen consortium, respectively. We analyzed a total of 166 bacterial features at four taxonomic levels, including order, family, genus, and species. The inverse-variance-weighted (IVW), weighted median, MR-Egger, and simple median methods were applied to the MR analysis, and the robustness of the results were tested using a series of sensitivity analyses. We extracted IVs of gut microbiota with direct causal association with CRC for SNP annotation to identify the genes in which these genetic variants were located to reveal the possible host gene-microbiome associations in CRC patients.

**Results:**

The findings from our MR analysis based on CRC-associated GWAS datasets from AGWAS revealed causal relationships between 6 bacterial taxa and CRC at a locus-wide significance level (P < 1 × 10^–5^). The IVW method found that family *Porphyromonadaceae*, genera *Anaerotruncus*, *Intestinibacter*, *Slackia*, and *Ruminococcaceae* UCG004, and species *Eubacterium coprostanoligenes* group were positively associated with CRC risk, which was generally consistent with the results of other complementary analyses. The results of a meta-analysis of the MR estimates from the AGWAS and the FinnGen datasets showed that family *Porphyromonadaceae* and genera *Slackia*, *Anaerotruncus*, and *Intestinibacter* replicated the same causal association. Sensitivity analysis of all causal associations did not indicate significant heterogeneity, horizontal pleiotropy, or reverse causal associations. We annotated the SNPs at a locus-wide significance level of the above intestinal flora and identified 24 host genes that may be related to pathogenic intestinal microflora in CRC patients.

**Conclusion:**

This study supported the causal relationship of gut microbiota on CRC and revealed a possible correlation between genes and pathogenic microbiota in CRC. These findings suggested that the study of the gut microbiome and its further multi-omics analysis was important for the prevention and treatment of CRC.

**Supplementary Information:**

The online version contains supplementary material available at 10.1186/s12967-023-04335-9.

## Introduction

Colorectal cancer (CRC) is a common malignancy of the digestive system that mainly originates from epithelial cells. It currently ranks third in incidence among common malignancies worldwide and is the second leading cause of tumor-related deaths [1, 2]. In recent years, the incidence of CRC has increased in many Asian countries including China [3]. It has become imperative to identify as many risk factors associated with CRC as possible for the prevention and treatment of CRC.

The human gastrointestinal tract hosts a large population of microorganisms that can interact with each other as well as with the intestinal microenvironment and other species in the environment. The relative abundance of certain gut microbiota may change under the influence of gene, drugs, and various metabolic and environmental factors, which can lead to a decrease in beneficial commensal flora and an increase in conditionally pathogenic and disease-causing bacteria [4], causing further changes in flora metabolism that can lead to disease in the intestine or in other target organs through a series of complex mechanisms. Several animal models have found an association between intestinal flora and CRC. In a study by Wong et al., feces from CRC patients and non-CRC patients were fed to healthy mice by gavage, and the results showed that the ratio of Th1 to Th17 cells, level of inflammatory markers, number of polyps, and proliferation levels of intestinal mucosal cells were significantly higher in mice fed feces from CRC patients compared to controls [5]. The association between intestinal flora and CRC has also been found in CRC patients with familial adenomatous polyposis (FAP), a precancerous condition of hereditary CRC. Dejea et al. found *E. coli* that formed biofilms as the predominant flora in surgically resected tissue from the colon of FAP patients, demonstrating that intestinal flora can form biofilms that induce upregulation of colonic epithelial interleukin 17 expression, causing abnormal alteration of colonic epithelial DNA, heterogeneous proliferation of epithelial cells, and subsequent progression to malignant tumor [6]. However, it is difficult to prove the causal association between gut microbiota and CRC by randomized controlled trials due to confounding factors such as diet, lifestyle, and the underdeveloped technology used in fecal transplantation experiments. In addition, recent studies have found a correlation between abnormal expression of genes related to CRC occurrence and the abundance of pathogenic bacteria [7, 8]. However, most studies have focused only on the association between a limited number of genes and gut microbes or specific bacteria [9, 10]. Therefore, the association of host genes with the gut microbiome in CRC needs to be further discovered and studied.

Mendelian randomization (MR) uses genetic variants in non-experimental data to infer the causal effect of an exposure on an outcome. The idea of MR is to use genome-wide association studies (GWAS) to obtain single-nucleotide polymorphisms (SNPs) that exhibit strong correlations with specific outcomes that can serve as a tool to infer causal associations between exposure factors and outcomes. These SNPs can be used to test for causal associations between exposure factors and outcomes while avoiding the effects of confounding factors because they are based on random Mendelian genetic variation. Biological genotypes are formed by random assignment during meiosis, a process that is generally not influenced by external factors. We therefore conducted an MR study to evaluate the causal association of gut microbiota on CRC. Annotation of the SNPs of the intestinal flora validated by MR analysis can find associated genes.

## Methods

### Data sources

We obtained SNPs associated with gut microbial abundance from the MiBioGen consortium’s GWAS study, which included 25 cohorts of 18,340 subjects from countries including the United States, Italy, and South Korea, and which focused on identifying genetic loci that influence the relative abundance of gut microbes by analyzing the 16SrRNA sequencing profiles of their subjects [11]. We obtained a dataset of genetic variants associated with CRC from a large GWAS study of East Asian populations, which included three cohorts with a total of 6692 CRC patients and 27,278 controls [12]. In addition, we obtained the CRC risk-related dataset from the FinnGen consortium for validation, which included 7427 CRC patients and 25,600 controls (Table 1) [13]. The GWAS studies selected for this MR analysis were ethically approved, and materials such as informed consent forms were available in the supplemental materials of the respective original publications.

**Table 1:** Detailed information of studies and datasets used for analyses

Data source	Phenotype	Sample size	Cases	Population	Adjustment
MiBioGen consortium	Gut microbial	18,340	–	The United States, Canada, Israel, South Korea, Germany, Denmark, the Netherlands, Belgium, Sweden, Finland and the United Kingdom	Age, sex, technical covariates, and genetic principal components
AGWAS	CRC	33,970	6692	Asian	Age, sex
FinnGen	CRC	33,027	7427	European	Age, sex

### Study design

**Fig. 1:**
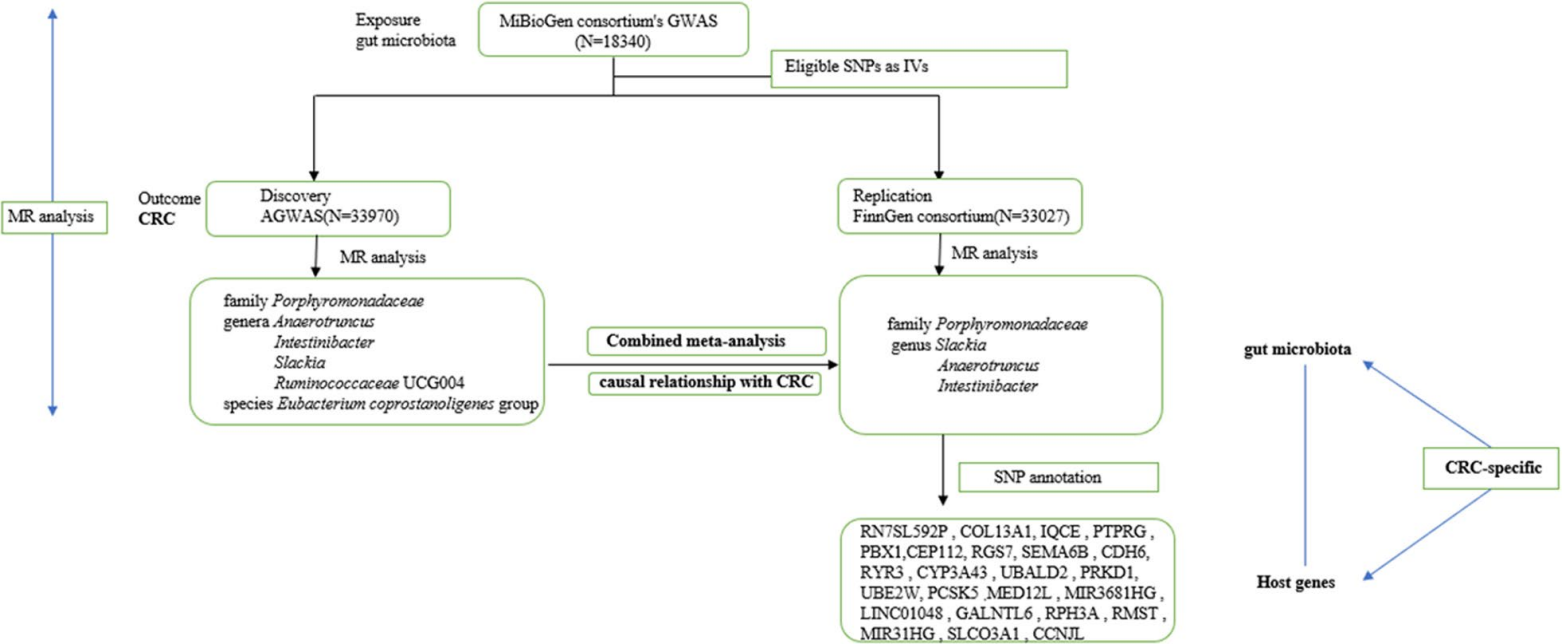
Overview of study design

**Fig. 2:**
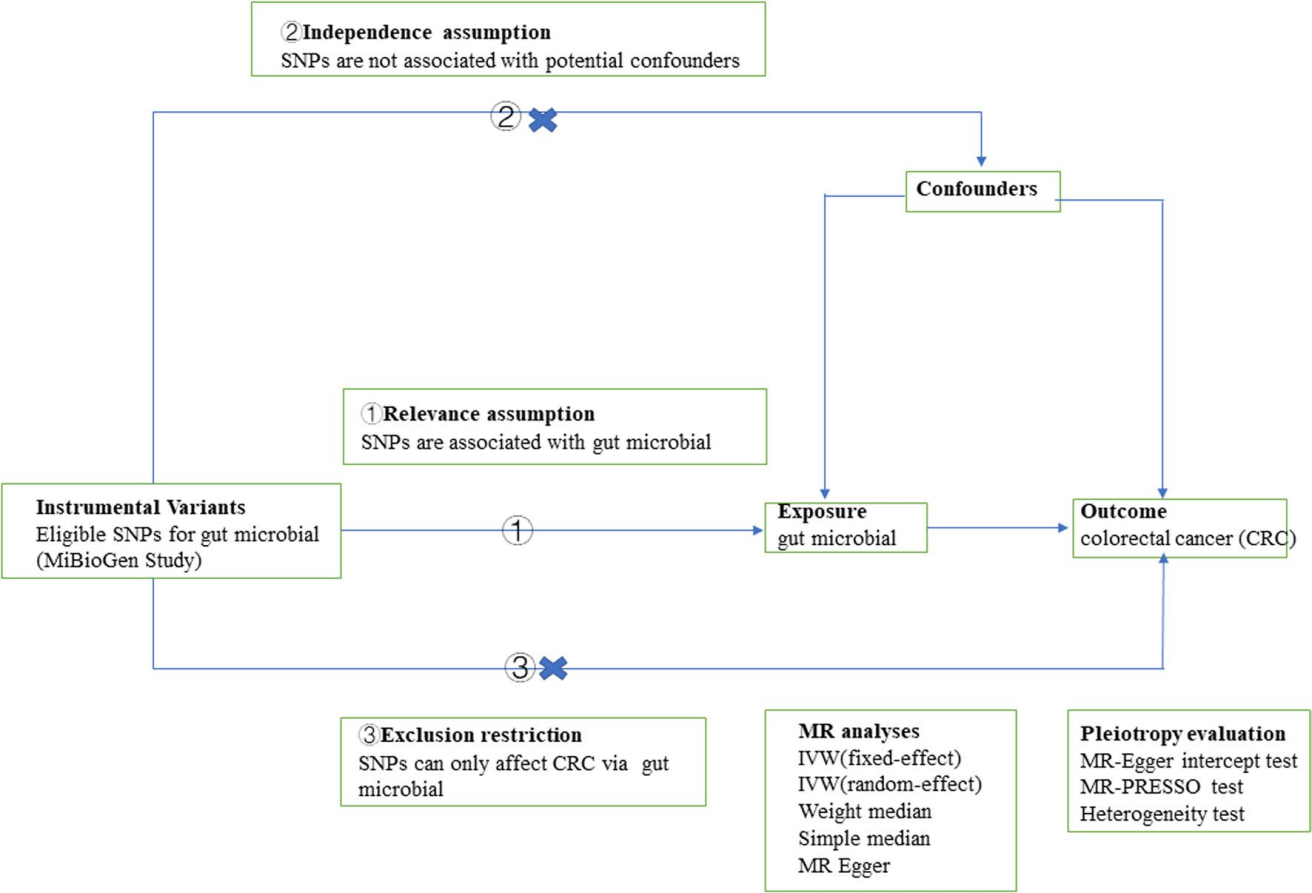
Schematic diagram of the present Mendelian randomization study

Our overall study design is shown in Fig. 1. We screened eligible SNPs from the GWAS dataset of the MiBioGen consortium using specific criteria as instrumental variables (shown in 2.3) for the gut microbiota. As shown in the Fig. 2, our MR study design satisfied the three necessary assumptions [14], and also followed the requirements of STROBE-MR [15] (Additional file 2: Table S1).

### Instrument selection

**Fig. 3:**
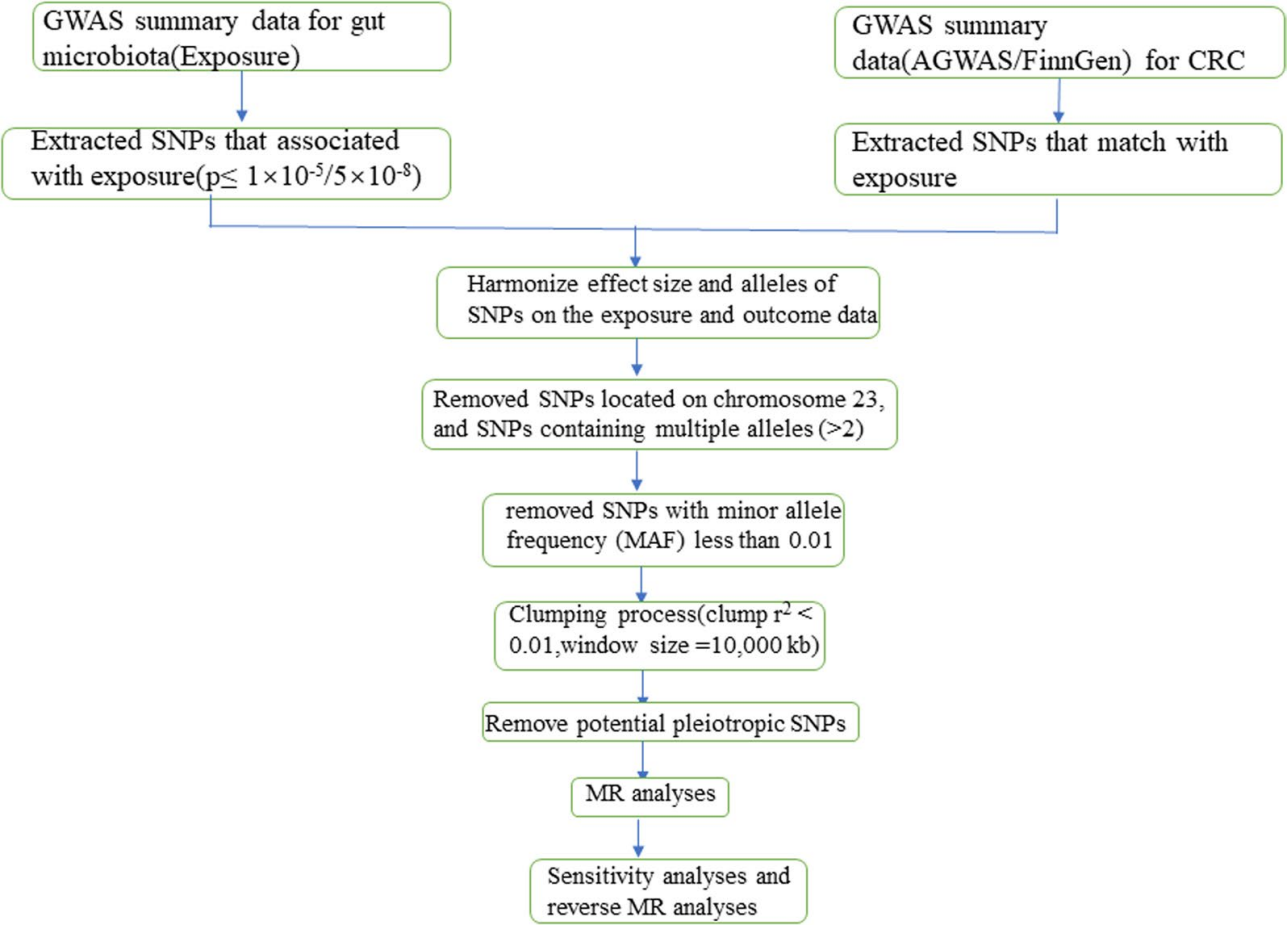
The whole workflow of MR analysis

First, we screened for SNPs associated with bacterial abundance from the GWAS study at the locus-wide significance level (P < 1 × 10^–5^) for each bacterial taxa at four taxonomic levels: order, family, genus, and species. Second, we screened and removed SNPs located on chromosome 23 and also removed SNPs containing multiple alleles (> 2) to avoid unwanted effects on our MR analysis results. Third, we removed SNPs with a minor allele frequency (MAF) of less than 0.01. Fourth, we used samples from the 1000 Genomes European Project as a reference to examine the linkage disequilibrium (LD) between instrumental variables (IVs), following the criteria of r^2^ < 0.01 and window size > 10,000 kb, thus avoiding the effect of LD between IVs. Fifth, some IVs may be strongly correlated (P < 5 × 10^–8^) with confounders or outcome events, referred to as horizontal pleiotropy, and the reliability of the results would be affected if these SNPs were included as instrumental variables for MR analysis [16]. Therefore, we obtained SNPs significantly associated with confounding characteristics (such as BMI and age) using PhenoScanner to preliminarily exclude the effect of horizontal pleiotropy. As a result, we did not detect SNPs with strong correlations with other confounding factors. Finally, we used SNPs that met all the above criteria as IVs for downstream MR analysis. We also screened for SNPs associated with gut microbial abundance from the GWAS study at a genome-wide significance level (P < 5 × 10^–8^) to include as IVs to make the analysis more comprehensive. The screening process for instrumental variables is shown in Fig. 3.

### Efficacy estimation of instrumental variables

The regression R^2^ value is often used in MR studies as a measure of how much the variance in the exposure outcome can be explained by the IVs. It is calculated as R^2^ = 2 × EAF × (1 − EAF) × beta^2^/(2 × EAF × (1 − EAF) × beta^2^) + 2 × EAF × (1 − EAF) × se × N × beta^2^ [17, 18]. Weak IVs in MR studies can cause bias in the causal association between exposure factors and outcome events. The F-statistic, derived from the regression of exposure outcomes on instrumental variables, can respond to the degree of correlation between exposure factors and outcomes and detect weak IVs. It is used to represent the degree of bias when estimating causal associations and is calculated using the formula F = R^2^ × (N − 2)/(1 − R^2^), where N represents the sample size of the exposed data [19]. An F-statistic less than 10 indicates the presence of weakly predictive instruments. This is derived from the observation that when F < 10, the bias of the IV estimate is more than 10% of the bias in the observational association estimate (relative bias > 1/10).

### Statistical analysis

We first obtained eligible SNPs as IVs using the process outlined above. For bacterial taxa containing only one IV, we used the Wald ratio for MR analysis. For bacterial taxa containing multiple IVs, we used the inverse-variance-weighted (IVW) approach as the main analysis method to examine the correlation between bacterial taxa and CRC. The IVW method is commonly used for obtaining variant-specific causal estimates, and can combine the effect values of multiple IVs into one estimate and provide a more accurate analysis of the causal relationships among variables. We also used the weighted median method, MR-Egger, simple median method, and MR-PRESSO as complementary analysis methods. The weighted median method is characterized by consistent results even when the weight of invalid IVs reach 50% (or < 50%) [20]. The MR-Egger method has relatively low statistical power [21], similar to the IVW method, except that the regression model contains an intercept term θ0 and the p-value of this intercept term can help identify horizontal pleiotropy [22]. We also applied the MR-PRESSO global test to detect horizontal pleiotropy, which is implemented using a weighted regression of all the genetic variants and then computing a residual sum of squares (RSS). Each IV would be removed in turn and the corresponding RSS value would be calculated. If the RSS value decreased significantly from the previous iteration and reached statistical significance (p < 0.05), it would suggest that the SNP exhibited horizontal pleiotropy. We tested for outlier SNPs using the MR-PRESSO outlier test and recalculated the estimates after removing any outliers, thus avoiding pleiotropic effects on our MR analysis [23].

We detected potential reverse causal associations between SNPs associated with the gut microbiota and CRC using the MR Steiger Filtering Test [24]. We used a series of sensitivity analyses to test the robustness of the results. We quantified heterogeneity by calculating Cochran’s Q statistic, which considers a result to be heterogeneous if the p-value is less than 0.05 [25]. The I^2^ statistic can also be used to quantify the degree of heterogeneity, and is calculated as I^2^ = (Q − Q_df)/Q. It can be assumed that there is heterogeneity if I^2^ is greater than 25% [25, 26]. The results of the analysis, based on the random effects model of the IVW method, may be more reliable if there is a high degree of heterogeneity among SNPs [27]. We assessed the heterogeneity between variant-specific causal estimates using meta-analysis techniques and identified outliers using scatter and funnel plots. In addition, we performed Leave-one-out analysis on IVs, in which all IVs of bacterial taxa were removed one by one, and recalculated MR estimates using all remaining SNPs to examine the correlation between the gut microbiota and CRC.

We performed MR analysis with the FinnGen consortium dataset to verify the accuracy of our results and meta-analyzed the MR estimates from the FinnGen and MiBioGen datasets. We used the mRnd online tool to calculate statistical power [28], which represents the ability to detect a particular magnitude of causal effect in a given sample size and should generally be greater than 80% to have confidence in the results. All statistical analyses were performed using the TwoSampleMR [29] and MR-PRESSO packages [23] in R4.2.0 [30].

### SNP annotation

The online network tool was used for SNP annotation [31]. g:SNPense maps a list of human SNP rs-codes to gene names, receives chromosomal coordinates and predicted variant effects. Mapping is enabled only for variants that overlap with at least one protein coding Ensembl gene. All underlying data are retrieved from the Ensembl Variation data.

## Results

### Instrumental variables selection

11,237 SNPs at the locus-wide significance level (P < 1 × 10^–5^) and 1035 SNPs at the genome-wide significance level (P < 5 × 10^–8^) were selected based on 166 bacterial features in the MiBioGen consortium. After identifying and removing SNPs in LD, the remaining 2271 SNPs at the locus-wide significance level and 12 SNPs at the genome-wide significance level were used as IVs. We extracted the effect allele, other allele, beta, SE, and p-value of these SNPs for MR analysis.

### Mendelian randomization analysis

#### Locus-wide significance level

**Fig. 4:**
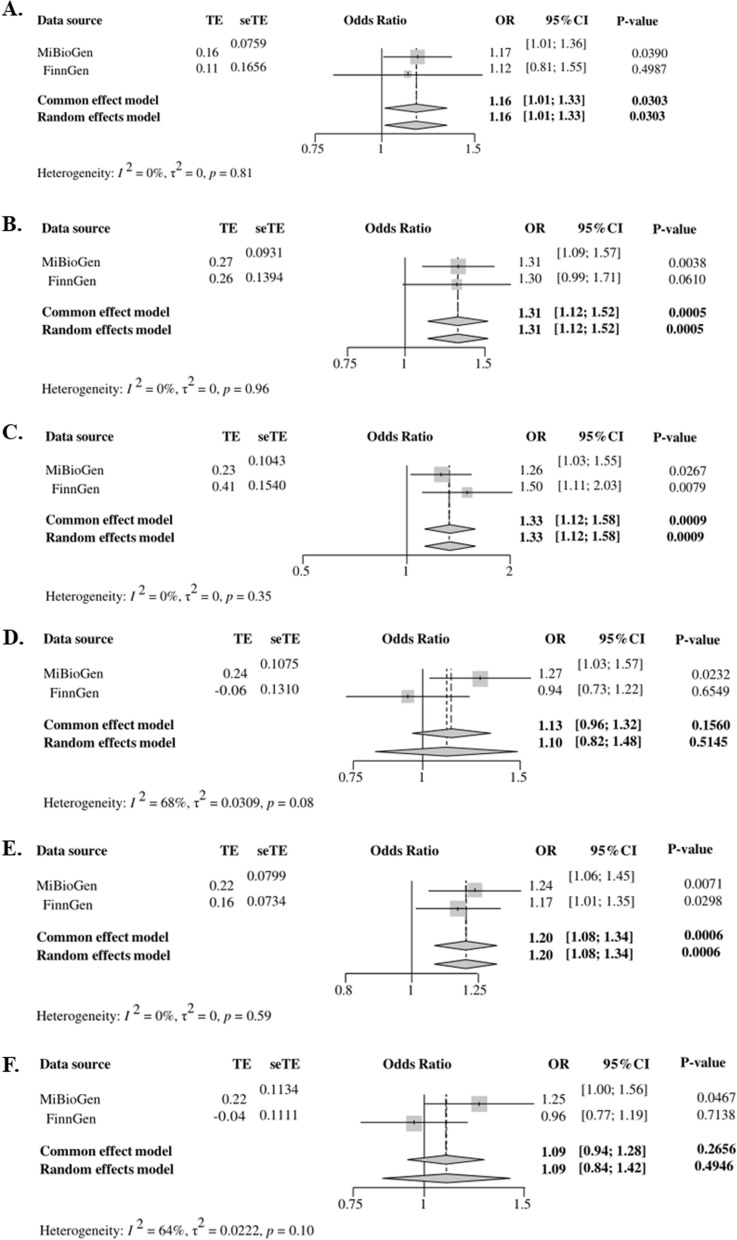
Association of genetically predicted Gut Microbiome with risk of CRC and combined MR estimates from both AGWAS and FinnGen databases by meta-analysis **A** genus Anaerotruncus, **B** genus Intestinibacter, **C** family Porphyromonadaceae, **D** genus RuminococcaceaeUCG004, **E** genus Slackia, **F** species *Eubacterium coprostanoligenes* group

The results of the IVW analysis showed that the family *Porphyromonadaceae* (OR = 1.26, 95% CI 1.03–1.55, P = 0.0267), genera *Anaerotruncus* (OR = 1.17, 95% CI 1.01–1.36, P = 0.0390), *Intestinibacter* (OR = 1.31, 95% CI 1.09–1.57, P = 0.0038), *Slackia* (OR = 1.24, 95% CI 1.06–1.45, P = 0.0071), and *Ruminococcaceae* UCG004 (OR = 1.27, 95% CI 1.03–1.57, P = 0.0232), and species *Eubacterium coprostanoligenes* group (OR = 1.25, 95% CI 1.00–1.56, P = 0.0467) exhibited significant causal associations with CRC risk. The results of weighted median method showed that the genus *Intestinibacter* (OR = 1.28, 95% CI 1.00–1.64, P = 0.0520) significantly increased the risk of CRC. According to the results of the simple median method, genus *Intestinibacter* (OR = 1.39, 95% CI 1.08–1.78, P = 0.0093) and species *Eubacterium coprostanoligenes* group (OR = 1.62, 95% CI 1.14–2.30, P = 0.0073) were positively associated with CRC risk, which was consistent with the results of the IVW analysis. The MR estimates from supplementary analysis all supported their negative effect on CRC (Table 2). Details on the SNPs used as bacterial features are shown in Additional file 2: Table S2. The F-statistics of the SNPs were all greater than 10, indicating no weak IVs were included. MR analysis based on the FinnGen database showed that family *Porphyromonadaceae* (OR = 1.50, 95% CI 1.11–2.03, P = 0.0079) and genus *Slackia* (OR = 1.17, 95% CI 1.02–1.36, P = 0.0298) were risk factors for CRC (Table 2). We combined MR estimates from both the AGWAS and FinnGen databases by meta-analysis and found that genus *Anaerotruncus* (OR = 1.16, 95% CI 1.01–1.33, P = 0.0303) and genus *Intestinibacter* (OR = 1.31, 95% CI 1.12–1.52, P = 0.0005) were positively associated with CRC. However, we found no associations between genus *Ruminococcaceae* UCG004 (OR = 1.13, 95% CI 0.96–1.32, P = 0.1560) and species *Eubacterium coprostanoligenes* group (OR = 1.09, 95% CI 0.94–1.28, P = 0.2656) with CRC. In summary, we found that family *Porphyromonadaceae*, genus *Slackia*, genus *Anaerotruncus*, and genus *Intestinibacter* all exhibited a significant causal association with CRC risk (Fig. 4).

**Table 2:** MR results of causal links between gut microbiome and CRC risk (P < 1 × 10^–5^)

Data source	Classification		Nsnp	Methods	OR (95% CI)	P-value
MiBioGen consortium	Family	Porphyromonadaceae.id.943	11	Inverse variance weighted (fixed effects)	1.26 (1.03, 1.55)	0.0267
				Inverse variance weighted (multiplicative random effects)	1.26 (1.02, 1.56)	0.0337
				Weighted median	1.25 (0.93, 1.67)	0.1337
				MR Egger	1.28 (0.83, 1.96)	0.2923
				Simple median	1.4 (0.94, 2.08)	0.1003
MiBioGen consortium	Genus	Anaerotruncus.id.2054	10	Inverse variance weighted (fixed effects)	1.17 (0.96, 1.42)	0.1121
				Inverse variance weighted (multiplicative random effects)	1.17 (1.01, 1.36)	0.0390
				Weighted median	1.14 (0.88, 1.49)	0.3184
				MR Egger	1.08 (0.63, 1.85)	0.7807
				Simple median	1.15 (0.87, 1.51)	0.3265
MiBioGen consortium		Intestinibacter.id.11345	10	Inverse variance weighted (fixed effects)	1.31 (1.09, 1.57)	0.0038
				Inverse variance weighted (multiplicative random effects)	1.31 (1.14, 1.5)	0.0001
				Weighted median	1.28 (1, 1.64)	0.0520
				MR Egger	1.06 (0.5, 2.26)	0.8849
				Simple median	1.39 (1.08, 1.78)	0.0093
MiBioGen consortium		Slackia.id.825	9	Inverse variance weighted (fixed effects)	1.24 (1.06, 1.45)	0.0071
				Inverse variance weighted (multiplicative random effects)	1.24 (1.01, 1.51)	0.0357
				Weighted median	1.15 (0.91, 1.44)	0.2363
				MR Egger	0.62 (0.24, 1.64)	0.3692
				Simple median	1.14 (0.88, 1.48)	0.3161
MiBioGen consortium		RuminococcaceaeUCG004.id.11362	9	Inverse variance weighted (fixed effects)	1.27 (1.03, 1.57)	0.0232
				Inverse variance weighted (multiplicative random effects)	1.27 (1.07, 1.51)	0.0053
				Weighted median	1.30 (0.99, 1.71)	0.0580
				MR Egger	2.09 (0.62, 7.13)	0.2754
				Simple median	1.32 (0.99, 1.74)	0.0563
MiBioGen consortium		Eubacteriumcoprostanoligenesgroup.id.11375	12	Inverse variance weighted (fixed effects)	1.25 (0.99, 1.58)	0.0583
				Inverse variance weighted (multiplicative random effects)	1.25 (1.00, 1.56)	0.0467
				Weighted median	1.28 (0.92, 1.79)	0.1387
				MR Egger	0.86 (0.31, 2.38)	0.7746
				Simple median	1.62 (1.14, 2.30)	0.0073
FinnGen	Family	Porphyromonadaceae.id.943	11	Inverse variance weighted (fixed effects)	1.50 (1.11, 2.03)	0.0079
				Inverse variance weighted (multiplicative random effects)	1.50 (1.18, 1.92)	0.0011
				Weighted median	1.44 (0.95, 2.2)	0.0892
				MR Egger	1.51 (0.7, 3.23)	0.3177
				Simple median	1.42 (0.93, 2.17)	0.1062
FinnGen	Genus	Anaerotruncus.id.2054	10	Inverse variance weighted (fixed effects)	1.12 (0.81, 1.55)	0.4987
				Inverse variance weighted (multiplicative random effects)	1.12 (0.72, 1.73)	0.6149
				Weighted median	0.91 (0.56, 1.49)	0.7151
				MR Egger	1.64 (0.38, 7.03)	0.5247
				Simple median	0.89 (0.55, 1.45)	0.6506
FinnGen		Intestinibacter.id.11345	10	Inverse variance weighted (fixed effects)	1.30 (0.99, 1.71)	0.0610
				Inverse variance weighted (multiplicative random effects)	1.30 (0.98, 1.72)	0.0641
				Weighted median	1.27 (0.86, 1.88)	0.2207
				MR Egger	2.12 (0.6, 7.54)	0.2790
				Simple median	1.35 (0.93, 1.97)	0.1110
FinnGen		Slackia.id.825	9	Inverse variance weighted (fixed effects)	1.17 (0.94, 1.46)	0.1557
				Inverse variance weighted (multiplicative random effects)	1.17 (1.02, 1.36)	0.0298
				Weighted median	1.24 (0.94, 1.64)	0.1302
				MR Egger	0.56 (0.16, 1.98)	0.4003
				Simple median	1.24 (0.92, 1.67)	0.1514
FinnGen		RuminococcaceaeUCG004.id.11362	9	Inverse variance weighted (fixed effects)	0.94 (0.73, 1.22)	0.6549
				Inverse variance weighted (multiplicative random effects)	0.94 (0.72, 1.23)	0.6687
				Weighted median	0.79 (0.56, 1.12)	0.1839
				MR Egger	0.86 (0.21, 3.5)	0.8353
				Simple median	0.79 (0.55, 1.14)	0.2153
FinnGen	Species	Eubacteriumcoprostanoligenesgroup.id.11375	12	Inverse variance weighted (fixed effects)	0.96 (0.71, 1.30)	0.7951
				Inverse variance weighted (multiplicative random effects)	0.96 (0.77, 1.19)	0.7138
				Weighted median	0.86 (0.57, 1.28)	0.4482
				MR Egger	1.05 (0.25, 4.35)	0.9509
				Simple median	0.90 (0.61, 1.34)	0.6130

The results of the MR steiger filtering test (Additional file 2: Table S3) did not reveal an inverse causal association between the bacterial taxa mentioned previously and CRC. There was no significant heterogeneity among SNPs for gut microbiome-CRC association, with low heterogeneity among all SNPs that served as IVs in all bacterial taxa (I^2^ < 25%, p Cochran’s Q > 0.01) except genus *Slackia* (I^2^ = 39%, p Cochran’s Q = 0.11) and genus *Anaerotruncus* (I^2^ = 45%, p Cochran’s Q = 0.06) (Table 3). Visualized scatter and funnel plots are shown in Additional file 1: Figs. S1–S12. Neither the Egger Intercept test nor the MR-PRESSO Global test detected significant horizontal pleiotropy. Similarly, the MR-PRESSO outlier test did not find any outlier SNPs that could lead to horizontal pleiotropy. The results of the Leave-one-out analyses showed no significant effect of individual SNPs on gut microbiome-CRC association. We had 97%, 99%, 72%, and 100% statistical power to detect ORs of 1.26, 1.24, 1.17, and 1.31 for associations of family *Porphyromonadaceae*, genus *Slackia*, genus *Anaerotruncus*, and genus *Intestinibacter* with CRC in the MiBioGen consortium, respectively. We had 100%, 99%, 60%, and 97% statistical power to detect the corresponding ORs of 1.41, 1.23, 1.07, and 1.24 in FinnGen.

**Table 3:** Evaluation of heterogeneity and directional pleiotropy using different methods

Data source	Classification	Bacterial taxas	Heterogeneity			Horizontal pleiotropy			
			I^2^ (%)	Cochran’s Q	P-value	Egger intercept	SE	P-value	MR-PRESSO global test p
MiBioGen consortium	Family	Porphyromonadaceae.id.943	8	10.89	0.37	0.00	0.02	0.94	0.5
MiBioGen consortium	Genus	Anaerotruncus.id.2054	0	5.33	0.80	0.01	0.02	0.76	0.797
MiBioGen consortium	Genus	Intestinibacter.id.11345	0	5.17	0.82	0.02	0.03	0.59	0.844
MiBioGen consortium	Genus	RuminococcaceaeUCG004.id.11362	0	5.31	0.72	− 0.04	0.05	0.44	0.779
MiBioGen consortium	Genus	Slackia.id.825	39	13.13	0.11	0.07	0.05	0.20	0.134
MiBioGen consortium	Species	Eubacteriumcoprostanoligenesgroup.id.11375	0	10.88	0.54	0.03	0.03	0.47	0.571
FinnGen	Family	Porphyromonadaceae.id.943	0	6.64	0.76	0.00	0.03	0.99	0.80
FinnGen	Genus	Anaerotruncus.id.2054	45	16.28	0.06	− 0.03	0.05	0.60	0.07
FinnGen	Genus	Intestinibacter.id.11345	2	9.22	0.42	− 0.04	0.05	0.46	0.45
FinnGen	Genus	RuminococcaceaeUCG004.id.11362	8	8.73	0.37	0.01	0.06	0.89	0.40
FinnGen	Genus	Slackia.id.825	0	3.41	0.91	0.07	0.06	0.28	0.92
FinnGen	Species	Eubacteriumcoprostanoligenesgroup.id.11375	0	5.51	0.90	− 0.01	0.04	0.91	0.90

#### Genome-wide statistical significance level

We first performed MR analysis of the 12 eligible SNPs in aggregate using IVW (OR = 1.01, 95% CI 0.88–1.15, P = 0.9062), the weighted median method (OR = 0.96, 95% CI 0.79–1.16, P = 0.6493), MR Egger (OR = 0.79, 95% CI 0.46–1.35, P = 0.4124), and the simple median method (OR = 1.12, 95% CI 0.93–1.35, P = 0.2284), none of which suggested that gut microbes were associated with CRC risk. Heterogeneity among IVs was low (p Cochran’s Q = 0.5720, I^2^ = 0), and the Egger intercept test and the MR-PRESSO Global Test results showed no significant levels of pleiotropy (Egger intercept p = 0.3820, MR-PRESOO global test p = 0.604). We did not find any bacterial taxa associated with CRC risk (Table 4**.**), We could not perform further tests for heterogeneity and pleiotropy because the number of IVs in each bacterial feature was less than 2.

**Table 4:** MR results of causal links between gut microbiome and CRC risk (P < 5 × 10^–8^)

Data source	Classification		Nsnp	Methods	OR (95% CI)	P-value	Heterogeneity				Horizontal pleiotropy		
							I^2^ (%)	Cochran’s Q	P-value	Egger intercept	SE	P-value	MR-PRESSO global test p
MiBioGen consortium	Total		12	Inverse variance weighted (fixed effects)	1.01 (0.88, 1.15)	0.906175	0	9.420471	0.571954	0.02709012	0.029626	0.38202	0.604
				Inverse variance weighted (multiplicative random effects)	1.01 (0.89, 1.14)	0.898653							
				Weighted median	0.96 (0.79, 1.16)	0.64935							
				MR Egger	0.79 (0.46, 1.35)	0.412427							
				Simple median	1.12 (0.93, 1.35)	0.228353							
MiBioGen consortium	Family	Peptostreptococcaceae	1	Wald ratio	4.74 (0.81, 27.82)	0.085157	–	–	–	–	–	–	–
MiBioGen consortium	Genus	Oxalobacter.id	1	Wald ratio	0.81 (0.60, 1.11)	0.198543	–	–	–	–	–	–	–
		Enterorhabdus.id	1	Wald ratio	1.11 (0.74, 1.66)	0.617075	–	–	–	–	–	–	–
		Erysipelatoclostridium	1	Wald ratio	1.20 (0.73, 1.99)	0.4751	–	–	–	–	–	–	–
		RuminococcaceaeUCG009	1	Wald ratio	1.36 (0.87, 2.12)	0.1755	–	–	–	–	–	–	–
		Bifidobacterium	1	Wald ratio	0.88 (0.59, 1.33)	0.5427	–	–	–	–	–	–	–
		RuminococcaceaeUCG013	1	Wald ratio	1.29 (0.41, 4.13)	0.6629	–	–	–	–	–	–	–
		Tyzzerella3	1	Wald ratio	0.92 (0.63, 1.34)	0.6599	–	–	–	–	–	–	–
		Allisonella	1	Wald ratio	0.9 (0.54, 1.5)	0.6729	–	–	–	–	–	–	–
MiBioGen consortium	Order	Bifidobacteriales	1	Wald ratio	0.88 (0.58, 1.33)	0.5427	–	–	–	–	–	–	–
MiBioGen consortium	Species	Eubacteriumcoprostanoligenesgroup	1	Wald ratio	0.8 (0.44, 1.46)	0.4634	–	–	–	–	–	–	–

### SNP annotation

We annotated the SNPs at a locus-wide significance level of the four intestinal flora and identified 24 host genes that may be related to pathogenic intestinal microflora in CRC patients (Table 5).

**Table 5:** SNP annotation of intestinal flora IVs

		id	chr	Start	End	Strand	Gene_ids	Gene_names
Family	Porphyromonadaceae	rs10119172		− 1	− 1		ENSG00000264615	RN7SL592P
		rs1029811		− 1	− 1		–	–
		rs10762312	10	69,812,107	69,812,107	+	ENSG00000197467 ENSG00000289193	COL13A1 –
		rs10858364		− 1	− 1		–	–
		rs12700163	7	2,609,042	2,609,042	+	ENSG00000106012	IQCE
		rs17065783	3	62,049,912	62,049,912	+	ENSG00000144724	PTPRG
		rs2066088	1	1.65E+08	1.65E+08	+	ENSG00000185630	PBX1
		rs2401072		− 1	− 1		–	–
		rs35233670	17	65,754,785	65,754,785	+	ENSG00000154240	CEP112
		rs35961441	1	2.41E+08	2.41E+08	+	ENSG00000226919 ENSG00000182901	– RGS7
		rs7330827		− 1	− 1		–	–
Genus	Slackia	rs1006200		− 1	− 1		–	–
		rs10409783	19	4,555,774	4,555,774	+	ENSG00000167680	SEMA6B
		rs11957560	5	31,268,861	31,268,861	+	ENSG00000113361 ENSG00000254138	CDH6 –
		rs12440440	15	33,749,695	33,749,695	+	ENSG00000198838	RYR3
		rs16894137		− 1	− 1		–	–
		rs35156985	7	99,854,092	99,854,092	+	ENSG00000021461	CYP3A43
		rs4492265	7	13,484,058	13,484,058	+	ENSG00000229618	–
		rs7710333	5	1.78E+08	1.78E+08	+	ENSG00000246596 ENSG00000290968	– –
		rs8901	17	76,270,929	76,270,929	+	ENSG00000185262	UBALD2
	Anaerotruncus	rs10150232	14	29,948,802	29,948,802	+	ENSG00000184304, ENSG00000257904	PRKD1 –
		rs11018566		− 1	− 1		–	–
		rs12056802	8	73,800,865	73,800,865	+	ENSG00000104343 ENSG00000258677	UBE2W –
		rs1272208	9	76,015,978	76,015,978	+	ENSG00000099139	PCSK5
		rs1431492	3	1.51E+08	1.51E+08	+	ENSG00000144893	MED12L
		rs4669806	2	12,060,626	12,060,626	+	ENSG00000224184	MIR3681HG
		rs6563550	13	37,484,276	37,484,276	+	ENSG00000230390	LINC01048
		rs7675045	4	1.72E+08	1.72E+08	+	ENSG00000174473	GALNTL6
		rs7963258	12	1.13E+08	1.13E+08	+	ENSG00000089169	RPH3A
		rs9347879		− 1	− 1		–	–
	Intestinibacter	rs10805326		− 1	− 1		–	–
		rs11109097	12	97,534,659	97,534,659	+	ENSG00000255794	RMST
		rs112879476		− 1	− 1		–	–
		rs16938435	9	21,502,924	21,502,924	+	ENSG00000171889	MIR31HG
		rs2098844		− 1	− 1		–	–
		rs2702387		− 1	− 1		–	–
		rs4327025	15	91,903,453	91,903,453	+	ENSG00000176463	SLCO3A1
		rs447950		− 1	− 1		–	–
		rs6875660	5	1.6E+08	1.6E+08	+	ENSG00000135083	CCNJL
		rs9348442		− 1	− 1		–	–

## Discussion

The human intestine is a diverse and nutrient-rich micro-ecological system, consisting of 100 trillion microbes mixed with digestive secretions, epithelial cells, and food-borne abiotic components. The intestinal flora regulates itself in healthy individuals to maintain the balance among the intestinal micro-ecological system while providing energy for the body through the digestion and absorption of food. The results from studies on intestinal flora in recent years have shown that changes in the structure, abundance, and function of intestinal flora are closely associated with many diseases including CRC [32]. There are significant differences in the number and species of intestinal flora between CRC patients and healthy people [33]. The degree of intestinal flora imbalance is positively correlated with the progression rate of CRC [34]. Several observational studies have found significant differences in gut flora composition between healthy patients and CRC patients at different stages of the disease from proliferative polyps and early cancer to metastatic malignancies, supporting the role of gut flora in the development of CRC [35]. However, other risk factors for CRC such as obesity, diet, lifestyle, and geography can also influence the composition of the gut microbiome. We thus do not know whether the alterations in the gut microbiome in CRC patients is secondary to the tumor or an active process that contributes to tumorigenesis. This potential reverse causal association prevents us from determining the direction of effect of the gut microbiome on CRC. In addition, previous studies have shown that microbiota can influence gene expression and that gene expression correlates with the abundance of gut microbiota, but studies on the association between broad gut microbiota and genes in CRC are limited [36, 37].We conducted this study to explore the causal association of the gut microbiome on CRC and identify possible associations between pathogenic bacteria and host genes in CRC. The results of the meta-analysis based on combining the MR estimates from the AGWAS and FinnGen datasets showed that the family *Porphyromonadaceae* and genera *Slackia*, *Anaerotruncus*, and *Intestinibacter* have a direct causal association on CRC.

The family *Porphyromonadaceae* contains a variety of genera such as *Parabacteroides*, *Odoribacter* and *Porphyromonas* that are rarely seen in healthy populations [38]. Zackular et al. constructed a mouse model that replicated the progression of CRC from chronic inflammation to heterogeneous hyperplasia to adenocarcinoma [39]. Their analysis of the gut microbiome composition of the mouse model showed a significantly elevated abundance of genus *Odoribacter* (belonging to family *Porphyromonadaceae*) [40]. Baxter et al. analyzed the gut microbial composition of the feces of several CRC patients (serving as the experimental group) and that of healthy individuals (serving as the control group), and then transplanted the feces into healthy mice to observe the differences in the number of tumors in the mice. The results showed a positive correlation between the genus *Parabacteroides* (belonging to family *Porphyromonadaceae*) and the incidence of CRC in the experimental group in contrast to the control group [41]. These studies suggest a pathogenic role of family *Porphyromonadaceae* in CRC, on the basis of which our study further revealed its causal association to CRC. However, because the family *Porphyromonadaceae* is relatively rare, research on its pathogenic mechanisms is limited and further studies on its role in the development of CRC are needed in the future.

For the genus *Anaerotruncus*, Loke et al. compared intestinal microbial composition and metabolomic differences between paired tumor tissue and normal tissue in 17 Asian CRC patients and found that the relative abundance of genus *Anaerotruncus* could influence steroid and terpene biosynthesis as well as bile metabolism, resulting in increased tumor-associated metabolites such as *S*-Adenosylmethionine (SAM) and *S*-Adenosyl-Homocysteine (SAH) [42]. Similarly, Satoh et al. identified significantly higher levels of SAM in tumor tissues of CRC patients compared to normal tissues [43]. Loke et al. revealed that gut microbiota dysbiosis caused local metabolic abnormalities at the primary tumor site, leading to significant upregulation of SAH levels [42]. Sibani et al. found that SAM and SAH levels were positively correlated with tumor number in animal models and could be used as a measure of abnormal cell transformation [44]. In addition, *Anaerotruncus* stimulates an increase in lipopolysaccharides (LPS) in humans which can disrupt the integrity of gastrointestinal epithelial cells and lead to impaired intestinal mucosal barrier function. Upregulated LPS promotes the release of pro-inflammatory cytokines and inhibits tight junction proteins, increasing oxidative stress and abnormal differentiation of colorectal epithelial cells [45, 46]. Enterotoxigenic *Bacteroides fragilis* (ETBF) is a Gram-negative anaerobic bacterium and Liu et al. [47] found that increased abundance of ETBF was closely associated with colorectal cancer. ETBF can produce *B. fragilis* toxin (BFT), which when bound to intestinal mucosal epithelial receptors, can promote the activation of Wnt and NF-KB signaling pathways, facilitating cell proliferation and DNA damage, leading to abnormal cell transformation [48–51]. ETBF can also cause the release of reactive oxygen species from inflammatory cells and promote the expression of cytokines and chemokines, leading to DNA damage which in turn promotes the development of CRC. These findings suggest that the genus Anaerotruncus plays an important role in the pathogenesis of CRC and can influence host gene expression, which is consistent with our results. Therefore, we speculate that the altered relative abundance of the genus *Anaerotruncus* affects local metabolism, leading to increased levels of metabolites such as SAM and SAH, which in turn cause host gene damage and results in the transformation of normal cells to tumors. Similarly, previous studies have found that genera *Slackia* and *Intestinibacter* are associated with CRC. Huo et al. compared the gut microbial composition of tissue samples from patients with and without CRC recurrence and found that the relative abundance of genus *Slackia* was significantly higher in patients with CRC recurrence than in patients without recurrence, suggesting that it is a potential biomarker for prognosis in CRC patients [52]. For genus *Intestinibacter*, many studies have found a significant increase in the abundance of this bacterium both in animal models with CRC and in the fecal and mucosal tissues of CRC patients [40, 41, 53]. For example, *Fusobacterium nucleatum* (FN) (belonging to genus *Intestinibacter*) can be involved in the development and metastasis of CRC through multiple mechanisms. Kostic et al. found that *Clostridium perfringens* suppressed anti-tumor immune responses by recruiting myeloid suppressor cells, tumor-associated macrophages, and regulatory T cells [54].

Previous observational studies have found an association between the gut microbiota and CRC, but the results cannot be used as evidence to support a direct causal association due to the influence of certain confounding factors such as the environment, diet. The significant advantage of our MR study is the selection of genetic variants significantly associated with the composition of the gut microbiota as IVs, which do not directly contribute to CRC and are not influenced by other risk factors for CRC. This means that any association between IVs with CRC must arise via the variant’s association with the gut microbiota, thus implying a causal effect of the gut microbiota on CRC.

Studies have shown that gut microbes can influence gene expression to regulate host physiology and even cause disease [36, 55, 56]. Similarly, related cellular experiments have found that certain gut microbes can affect gene expression in colonic epithelial cells [37], and that the relative abundance of certain pathogenic gut microbes correlates with the expression of known CRC pathogenic genes [7, 8], all of which reveal the important role of gut microbe-host gene interactions in the development of CRC. We identified 24 host genes that may be associated with the abundance of gut microbes in CRC-specific populations by SNP annotation, including the PCSK5 gene, which was consistent with the findings of Sambhawa Priya et al. [57], who identified CRC disease-specific host gene-microbiome associations using a multi-omics integration model approach different from ours, on the basis of which we found that this gene may be associated with the abundance of the genus Anaerotruncus. Liao et al. used weighted gene co-expression network analysis to reveal that MIR22HG may regulate PCSK5 and RP11-61I13.3 may act on CRC progression by regulating PCSK5 through sponge-like miRNAs [58].

However, there are still unavoidable limitations of the present MR study. First, our MR analysis based on IVs at the genome-wide statistical significance level (P < 5 × 10^–8^) do not identify any causal association of the gut microbiome on CRC. All causal associations revealed by our MR study were obtained based on IVs at the locus-wide significance level (P < 1 × 10^–5^), which may have an impact on the accuracy of the results. Second, the causal association of genus *Anaerotruncus* on CRC do not reach the desired statistical power threshold of 80%, so the correlation needs to be further clarified. Third, since detailed baseline characteristics of study subjects (e.g., age, tumor markers, tumor stage, etc.) were not provided in the GWAS study of CRC, we could not further investigate the effect of gut microbiome on different subgroups of the population. Fourth, although we identified possible gene-gut microbiome associations through SNP annotation, the diagnostic and prognostic value of the CRC-specific gut microbiome-host gene associations we identified remains to be validated by further clinical studies due to the limited number of available studies.

In conclusion, this MR study demonstrates that several gut microbes are positively associated with CRC risk and can serve as potential biomarkers, on the basis of which this study also identified possible gene-gut microbiome associations in CRC. We call for in vivo or in vitro experiments to investigate CRC-specific host gene-gut microbial abundance and metabolomic correlations based on multi-omics, thus revealing the pathogenic mechanisms of gut flora and exploring potential biomarkers, which are important to optimize the diagnosis and treatment of CRC in the future.

### Supplementary Information

**Additional file 1: Figure S1.** Forest plot (A), sensitivity analysis (B), scatter plot (C), and funnel plot (D) of the causal effect of family Porphyromonadaceae on CRC risk based on AGWAS. **Figure S2.** Forest plot (A), sensitivity analysis (B), scatter plot (C), and funnel plot (D) of the causal effect of genus Anaerotruncus on CRC risk based on AGWAS. **Figure S3.** Forest plot (A), sensitivity analysis (B), scatter plot (C), and funnel plot (D) of the causal effect of genus Intestinibacter on CRC risk based on AGWAS. **Figure S4.** Forest plot (A), sensitivity analysis (B), scatter plot (C), and funnel plot (D) of the causal effect of genus Slackia on CRC risk based on AGWAS. **Figure S5.** Forest plot (A), sensitivity analysis (B), scatter plot (C), and funnel plot (D) of the causal effect of genus RuminococcaceaeUCG004 on CRC risk based on AGWAS. **Figure S6.** Forest plot (A), sensitivity analysis (B), scatter plot (C), and funnel plot (D) of the causal effect of species *Eubacterium coprostanoligenes* group on CRC risk based on AGWAS. **Figure S7.** Forest plot (A), sensitivity analysis (B), scatter plot (C), and funnel plot (D) of the causal effect of family Porphyromonadaceae on CRC risk based on FinnGen. **Figure S8.** Forest plot (A), sensitivity analysis (B), scatter plot (C), and funnel plot (D) of the causal effect of genus Anaerotruncus on CRC risk based on FinnGen. **Figure S9.** Forest plot (A), sensitivity analysis (B), scatter plot (C), and funnel plot (D) of the causal effect of genus Intestinibacter on CRC risk based on FinnGen. **Figure S10.** Forest plot (A), sensitivity analysis (B), scatter plot (C), and funnel plot (D) of the causal effect of genus Slackia on CRC risk based on FinnGen. **Figure S11.** Forest plot (A), sensitivity analysis (B), scatter plot (C), and funnel plot (D) of the causal effect of genus RuminococcaceaeUCG004 on CRC risk based on FinnGen. **Figure S12.** Forest plot (A), sensitivity analysis (B), scatter plot (C), and funnel plot (D) of the causal effect of species *Eubacterium coprostanoligenes* group on CRC risk based on FinnGen.

**Additional file 2: Table S1.** STROBE-MR Checklist. **Table S2.** SNPs used as instrumental variables from gut microbiome and CRC GWASs (P < 1 × 10^–5^). **Table S3.** Results of MR Steiger direction test. **Table S4.** Power calculations in Mendelian randomization study.

## Data Availability

The original contributions presented in the study are included in the article/Additional files, further inquiries can be directed to the corresponding author/s.

## Abbreviations

CRC: Colorectal cancer
MR: Mendelian randomization
GWAS: Genome-wide association study
AGWAS: Asian Population Genome-wide association study
IVW: Inverse-variance-weighted
FAP: Familial adenomatous polyposis
SNP: Single-nucleotide polymorphisms
MAF: Minor allele frequency
LD: Linkage disequilibrium
SE: Standard error
EAF: Effect allele frequency
RSS: Residual sum of squares
SAM: S-Adenosylmethionine
SAH: *S*-Adenosyl-homocysteine
LPS: Lipopolysaccharide
ETBF: Enterotoxigenic *Bacteroides fragilis*
BFT: *B. fragilis* Toxin
