# Supplementary material for: Identification of host gene-microbiome associations in colorectal cancer patients using mendelian randomization
Source: J Transl Med. 2023 Aug 10;21:535. doi: 10.1186/s12967-023-04335-9 (PMC10416448; doi:10.1186/s12967-023-04335-9)
Supplement: Supplementary file 1 — Additional file 1: Figure S1. Forest plot (A), sensitivity analysis (B), scatter plot (C), and funnel plot (D) of the causal effect of family Porphyromonadaceae on CRC risk based on AGWAS. Figure S2. Forest plot (A), sensitivity analysis (B), scatter plot (C), and funnel plot (D) of the causal effect of genus Anaerotruncus on CRC risk based on AGWAS. Figure S3. Forest plot (A), sensitivity analysis (B), scatter plot (C), and funnel plot (D) of the causal effect of genus Intestinibacter on CRC risk based on AGWAS. Figure S4. Forest plot (A), sensitivity analysis (B), scatter plot (C), and funnel plot (D) of the causal effect of genus Slackia on CRC risk based on AGWAS. Figure S5. Forest plot (A), sensitivity analysis (B), scatter plot (C), and funnel plot (D) of the causal effect of genus RuminococcaceaeUCG004 on CRC risk based on AGWAS. Figure S6. Forest plot (A), sensitivity analysis (B), scatter plot (C), and funnel plot (D) of the causal effect of species Eubacterium coprostanoligenes group on CRC risk based on AGWAS. Figure S7. Forest plot (A), sensitivity analysis (B), scatter plot (C), and funnel plot (D) of the causal effect of family Porphyromonadaceae on CRC risk based on FinnGen. Figure S8. Forest plot (A), sensitivity analysis (B), scatter plot (C), and funnel plot (D) of the causal effect of genus Anaerotruncus on CRC risk based on FinnGen. Figure S9. Forest plot (A), sensitivity analysis (B), scatter plot (C), and funnel plot (D) of the causal effect of genus Intestinibacter on CRC risk based on FinnGen. Figure S10. Forest plot (A), sensitivity analysis (B), scatter plot (C), and funnel plot (D) of the causal effect of genus Slackia on CRC risk based on FinnGen. Figure S11. Forest plot (A), sensitivity analysis (B), scatter plot (C), and funnel plot (D) of the causal effect of genus RuminococcaceaeUCG004 on CRC risk based on FinnGen. Figure S12. Forest plot (A), sensitivity analysis (B), scatter plot (C), and funnel plot (D) of the causal effect of spec [file 12967_2023_4335_MOESM1_ESM.docx]

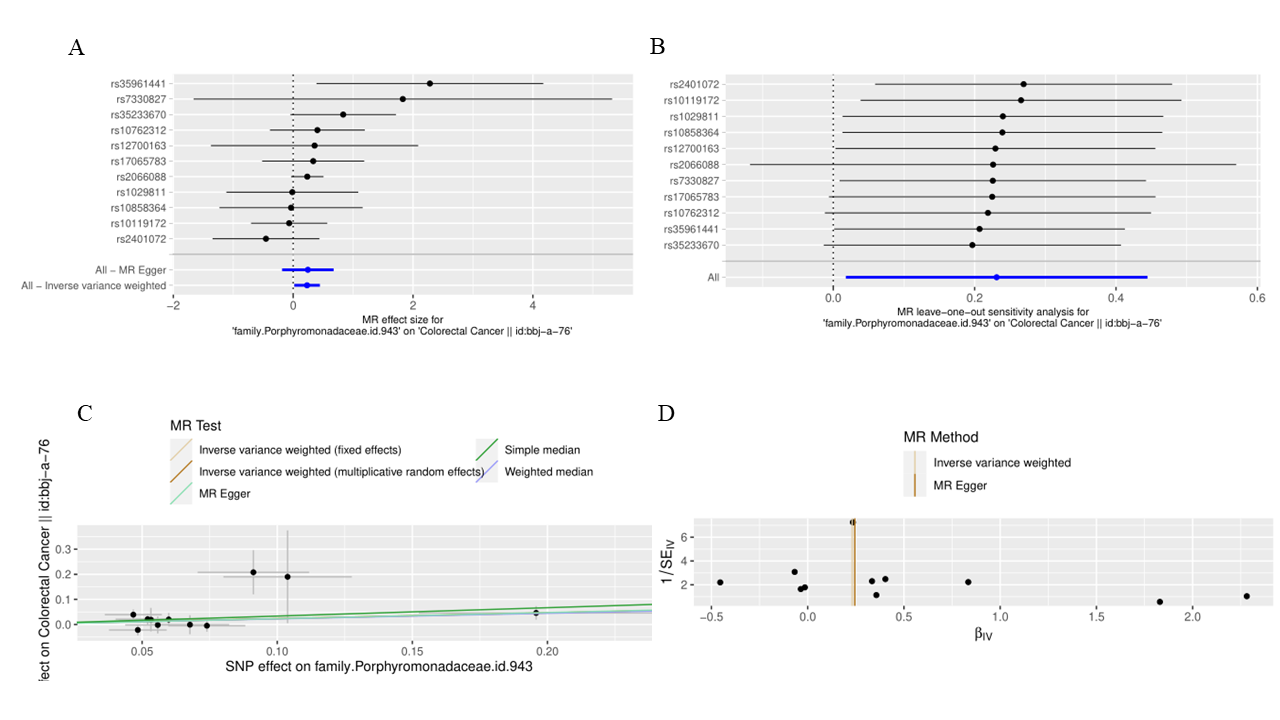


**Figure S1.** Forest plot (A), sensitivity analysis (B), scatter plot (C), and funnel plot (D) of the causal effect of family Porphyromonadaceae on CRC risk based on AGWAS.


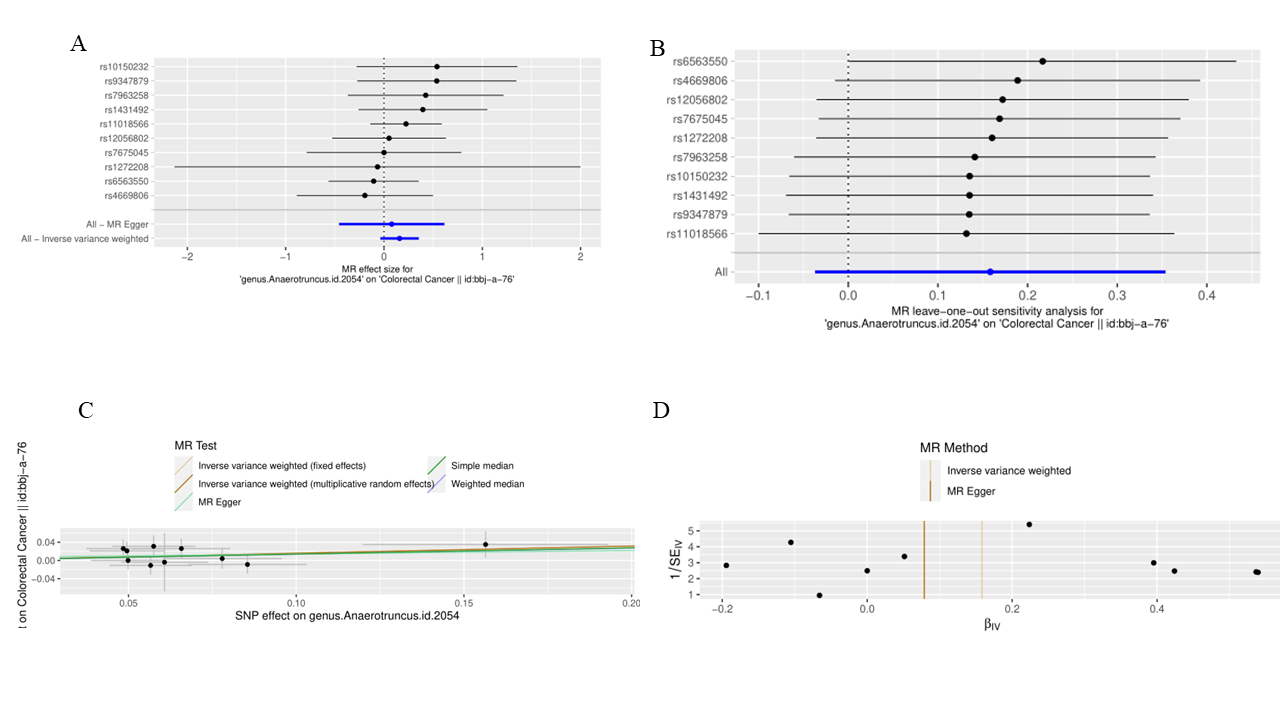


**Figure S2.** Forest plot (A), sensitivity analysis (B), scatter plot (C), and funnel plot (D) of the causal effect of genus Anaerotruncus on CRC risk based on AGWAS.


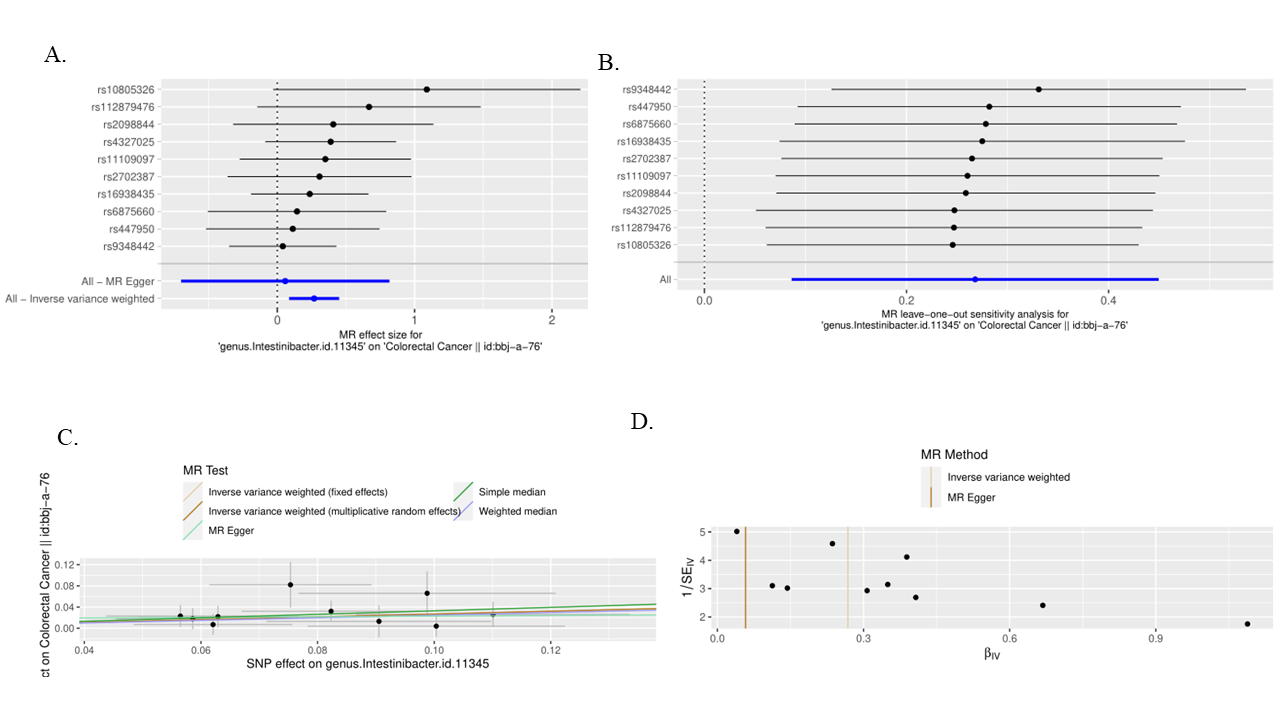


**Figure S3.** Forest plot (A), sensitivity analysis (B), scatter plot (C), and funnel plot (D) of the causal effect of genus Intestinibacter on CRC risk based on AGWAS.


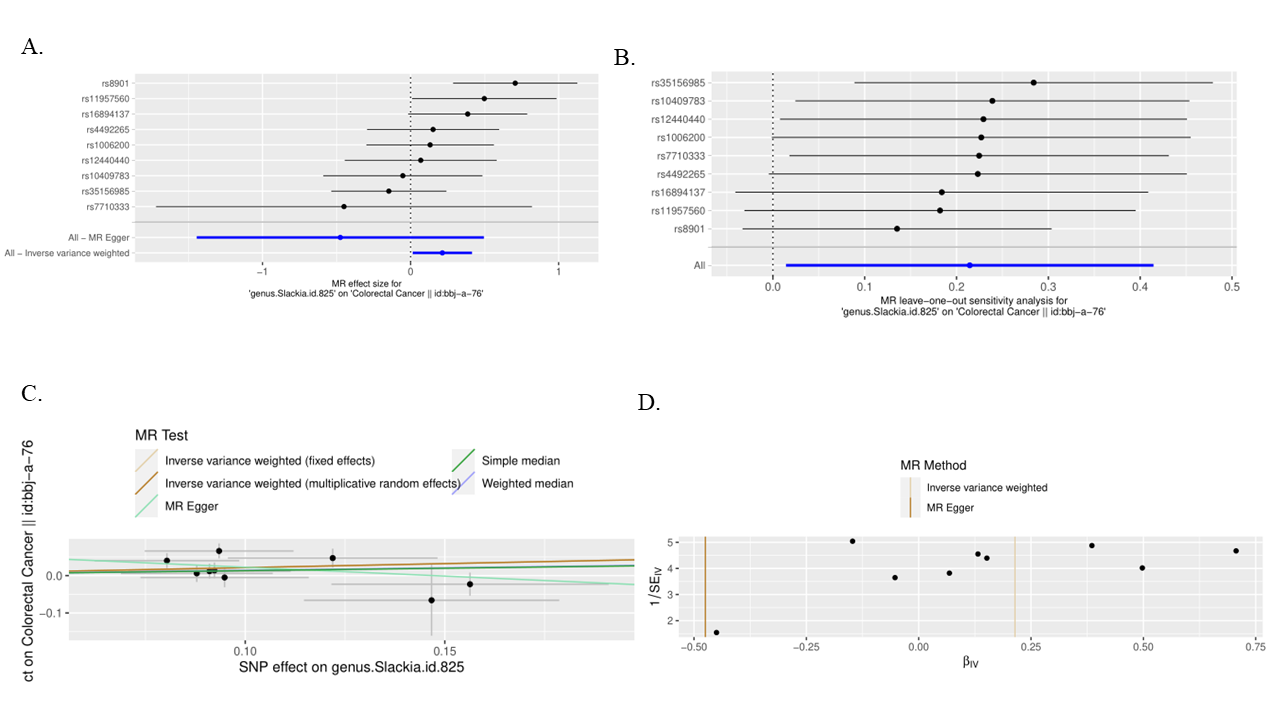


**Figure S4.** Forest plot (A), sensitivity analysis (B), scatter plot (C), and funnel plot (D) of the causal effect of genus Slackia on CRC risk based on AGWAS.


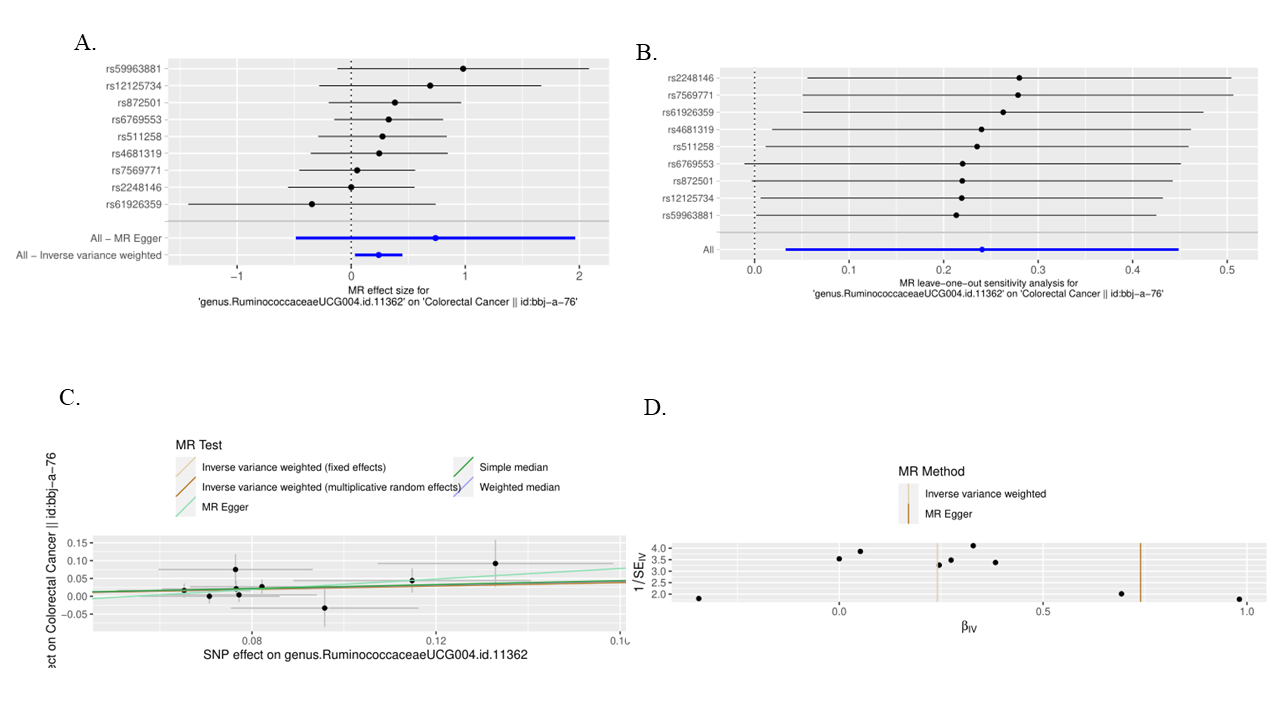


**Figure S5.** Forest plot (A), sensitivity analysis (B), scatter plot (C), and funnel plot (D) of the causal effect of genus RuminococcaceaeUCG004 on CRC risk based on AGWAS.


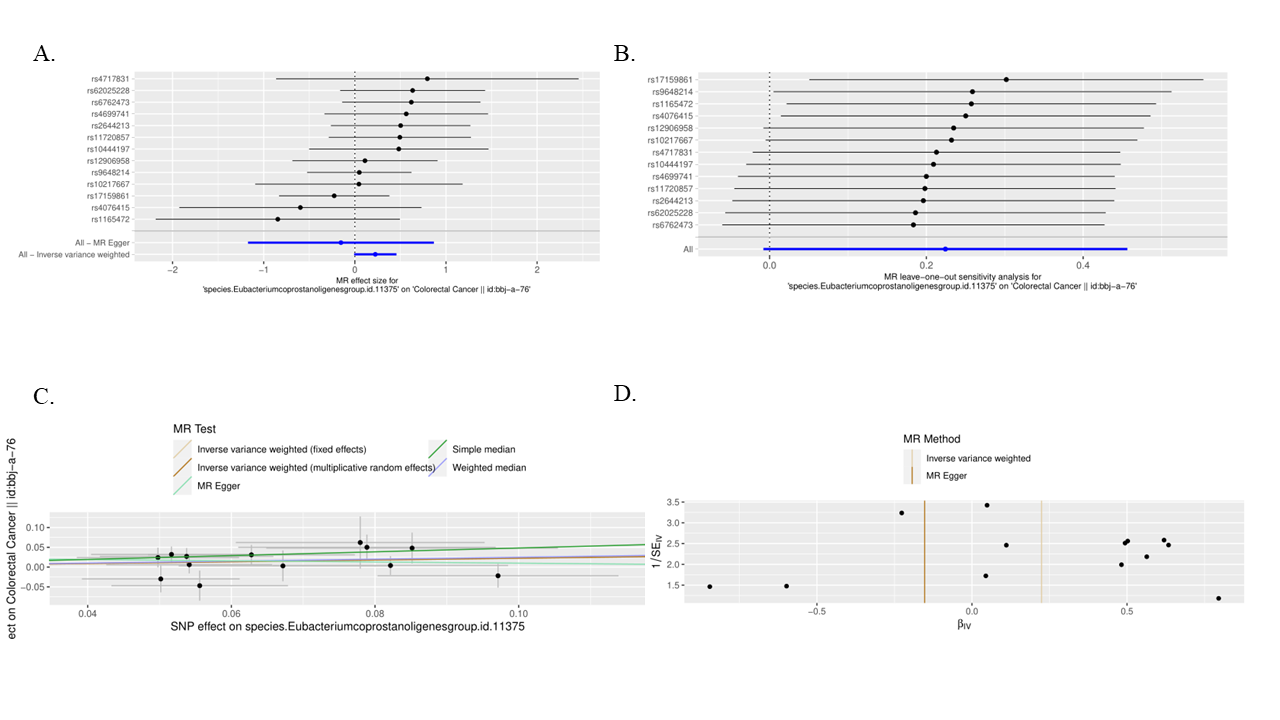


**Figure S6.** Forest plot (A), sensitivity analysis (B), scatter plot (C), and funnel plot (D) of the causal effect of species Eubacteriumcoprostanoligenesgroup on CRC risk based on AGWAS.


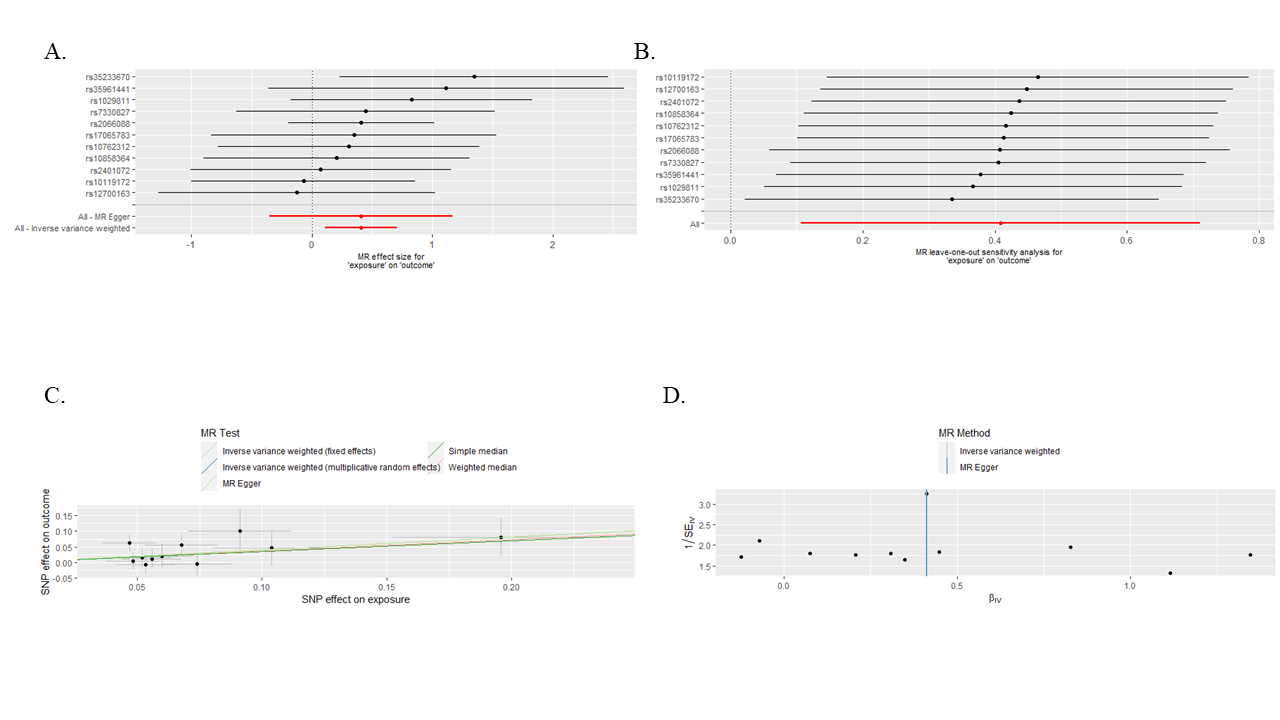


**Figure S7.** Forest plot (A), sensitivity analysis (B), scatter plot (C), and funnel plot (D) of the causal effect of family Porphyromonadaceae on CRC risk based on FinnGen.


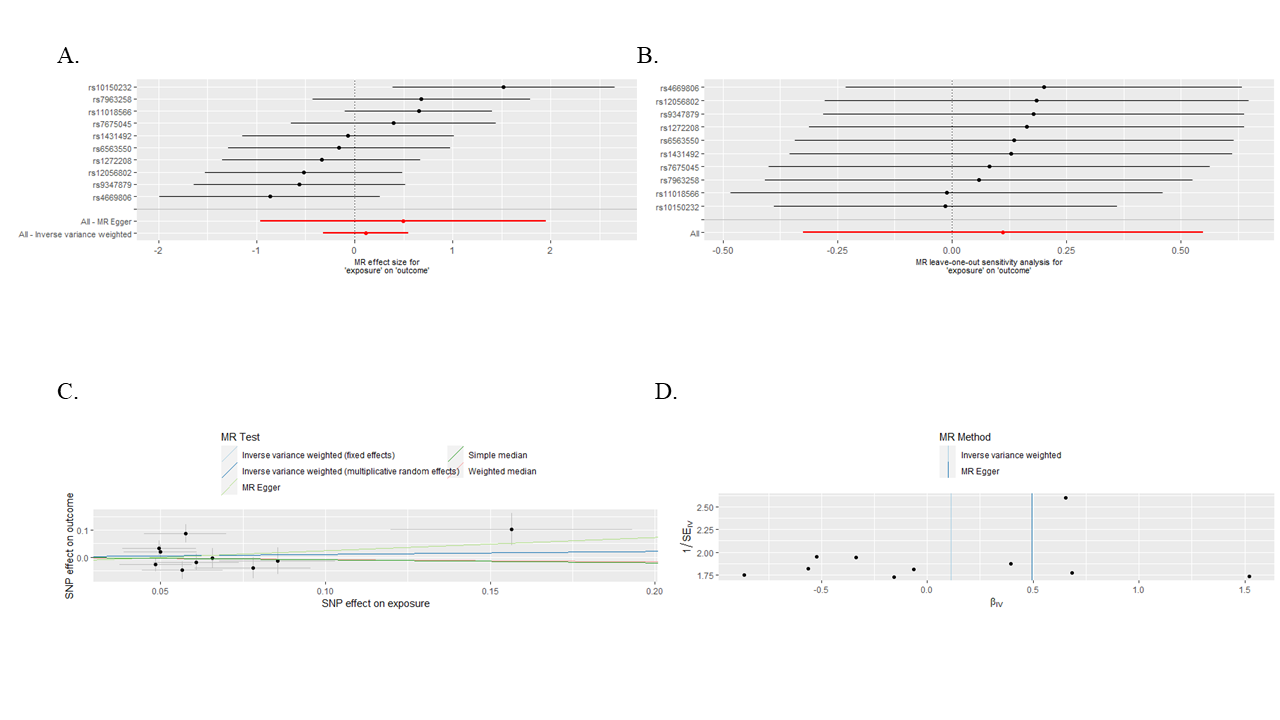


**Figure S8.** Forest plot (A), sensitivity analysis (B), scatter plot (C), and funnel plot (D) of the causal effect of genus Anaerotruncus on CRC risk based on FinnGen.


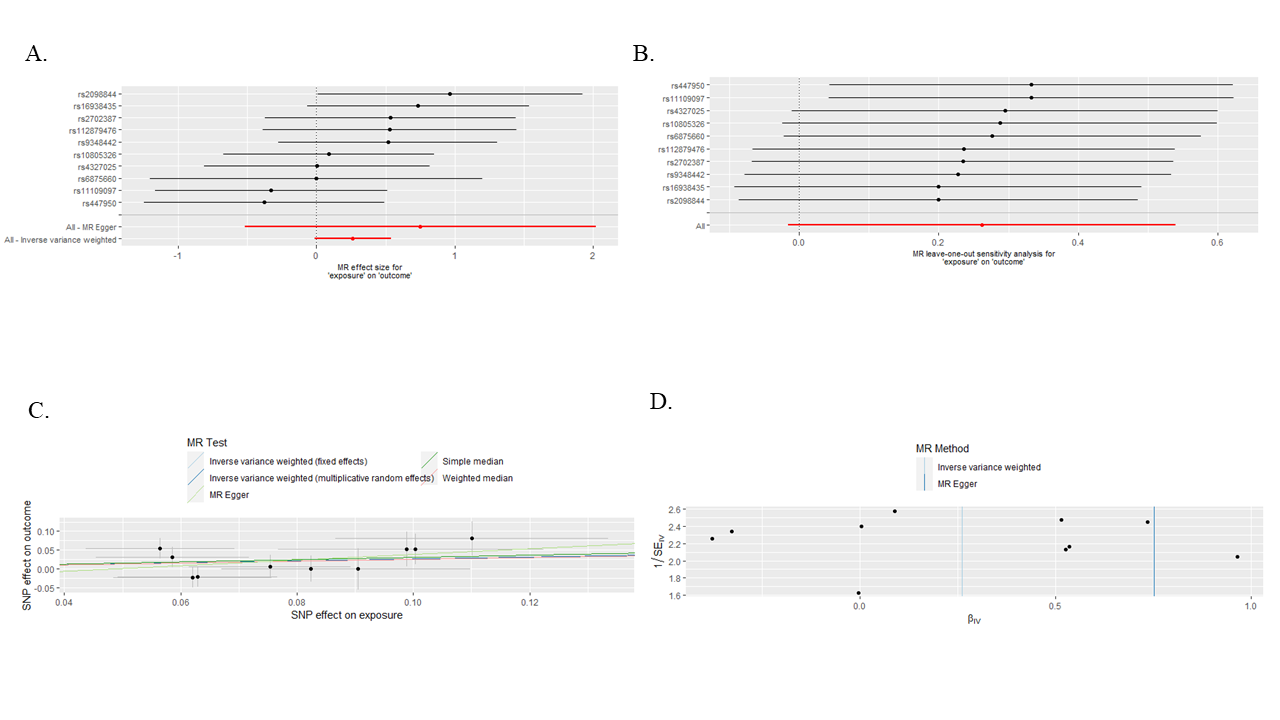


**Figure S9.** Forest plot (A), sensitivity analysis (B), scatter plot (C), and funnel plot (D) of the causal effect of genus Intestinibacter on CRC risk based on FinnGen.


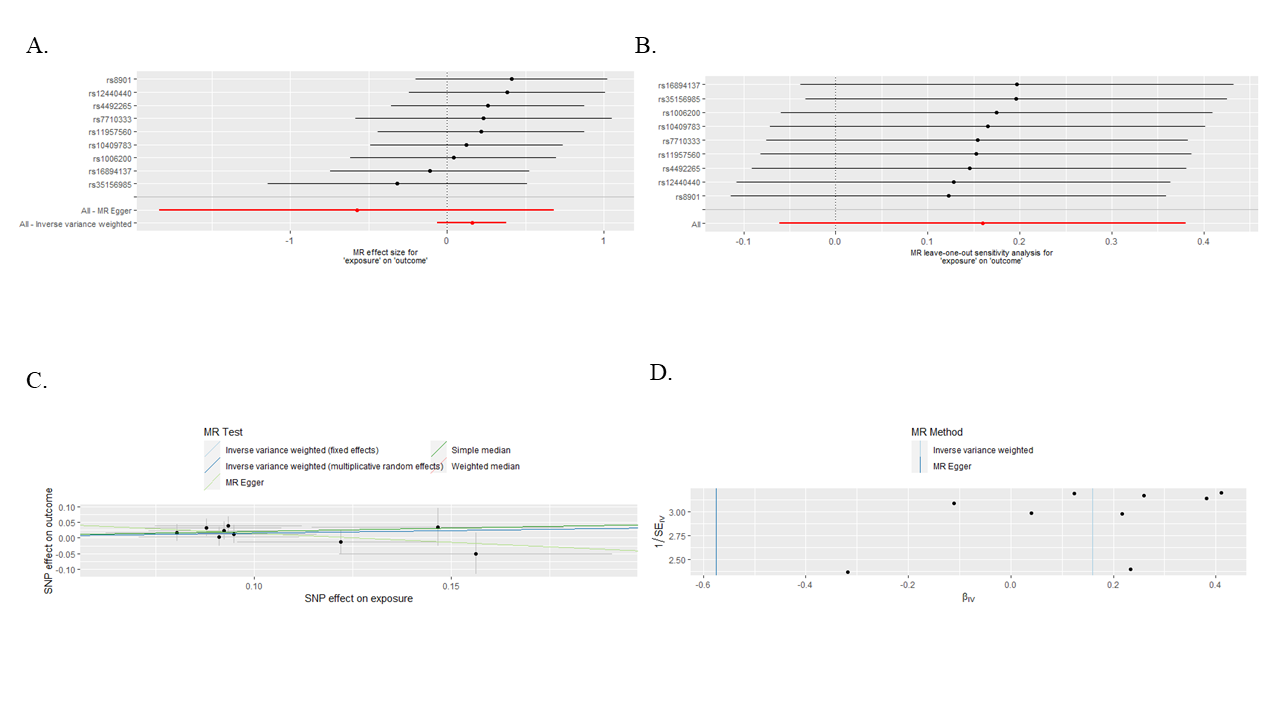


**Figure S10.** Forest plot (A), sensitivity analysis (B), scatter plot (C), and funnel plot (D) of the causal effect of genus Slackia on CRC risk based on FinnGen.


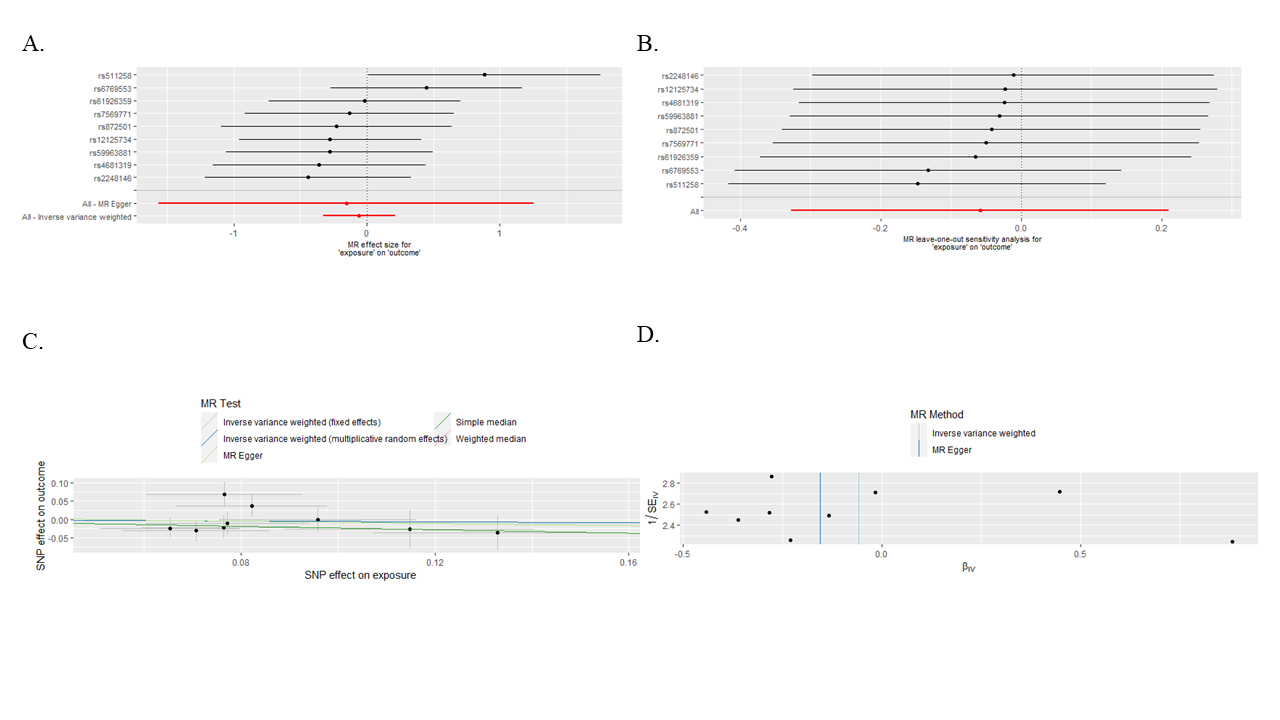


**Figure S11.** Forest plot (A), sensitivity analysis (B), scatter plot (C), and funnel plot (D) of the causal effect of genus RuminococcaceaeUCG004 on CRC risk based on FinnGen.


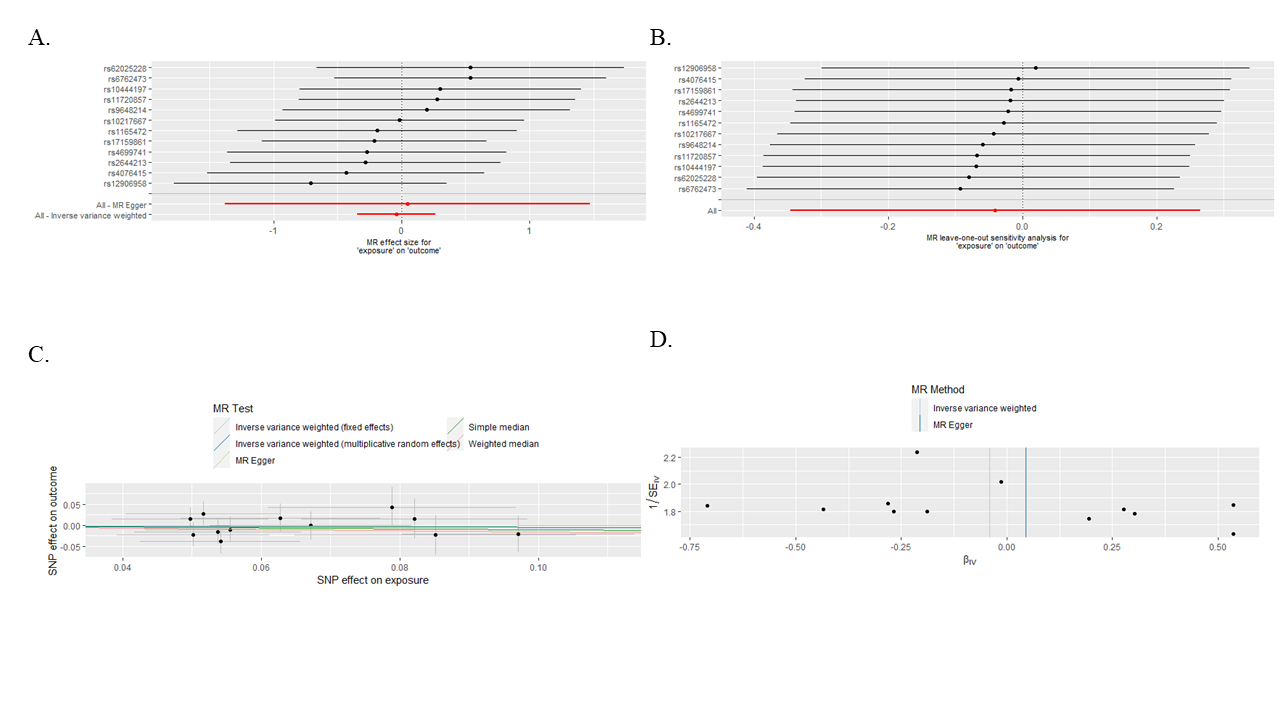


**Figure S12.** Forest plot (A), sensitivity analysis (B), scatter plot (C), and funnel plot (D) of the causal effect of species Eubacterium coprostanoligenes group on CRC risk based on FinnGen.
